# Integrative transcriptomic, proteomic, and machine learning approach to identifying feature genes of atrial fibrillation using atrial samples from patients with valvular heart disease

**DOI:** 10.1186/s12872-020-01819-0

**Published:** 2021-01-28

**Authors:** Yaozhong Liu, Fan Bai, Zhenwei Tang, Na Liu, Qiming Liu

**Affiliations:** 1 Department of Cardiovascular Medicine/Cardiac Catheterization Lab, Second Xiangya Hospital, Central South University, No. 139 Middle Renmin Road, Changsha, 410011 Hunan Province People’s Republic of China; 2 Department of Dermatology, Xiangya Hospital, Central South University, Changsha, Hunan Province People’s Republic of China

**Keywords:** Atrial fibrillation, Transcriptomic, Proteomic, Machine learning, Feature gene

## Abstract

**Background:**

Atrial fibrillation (AF) is the most common arrhythmia with poorly understood mechanisms. We aimed to investigate the biological mechanism of AF and to discover feature genes by analyzing multi-omics data and by applying a machine learning approach.

**Methods:**

At the transcriptomic level, four microarray datasets (GSE41177, GSE79768, GSE115574, GSE14975) were downloaded from the Gene Expression Omnibus database, which included 130 available atrial samples from AF and sinus rhythm (SR) patients with valvular heart disease. Microarray meta-analysis was adopted to identified differentially expressed genes (DEGs). At the proteomic level, a qualitative and quantitative analysis of proteomics in the left atrial appendage of 18 patients (9 with AF and 9 with SR) who underwent cardiac valvular surgery was conducted. The machine learning correlation-based feature selection (CFS) method was introduced to selected feature genes of AF using the training set of 130 samples involved in the microarray meta-analysis. The Naive Bayes (NB) based classifier constructed using training set was evaluated on an independent validation test set GSE2240.

**Results:**

863 DEGs with FDR < 0.05 and 482 differentially expressed proteins (DEPs) with FDR < 0.1 and fold change > 1.2 were obtained from the transcriptomic and proteomic study, respectively. The DEGs and DEPs were then analyzed together which identified 30 biomarkers with consistent trends. Further, 10 features, including 8 upregulated genes (CD44, CHGB, FHL2, GGT5, IGFBP2, NRAP, SEPTIN6, YWHAQ) and 2 downregulated genes (TNNI1, TRDN) were selected from the 30 biomarkers through machine learning CFS method using training set. The NB based classifier constructed using the training set accurately and reliably classify AF from SR samples in the validation test set with a precision of 87.5% and AUC of 0.995.

**Conclusion:**

Taken together, our present work might provide novel insights into the molecular mechanism and provide some promising diagnostic and therapeutic targets of AF.

## Background

Atrial fibrillation (AF) is the most common cardiac arrhythmia and is a leading cause of stroke, heart failure, and dementia [1]. AF currently affects over 30 million individuals worldwide [2], and this number is projected to grow dramatically over the next 20 years [3]. Despite > 100 years of basic and clinical research, the fundamental mechanisms of AF remain poorly understood.

Microarray expression analysis of atrial tissues can provide a global unbiased framework to characterize the transcriptional changes associated with AF. Advancement of high-throughput microarray technology is producing a large number of gene expression data, which are powerful tools for discovering and studying novel biomarkers for AF. Nonetheless, analysis based on high throughput data may face the dreaded ‘curse of dimensionality’. This refers to the phenomenon that the amount of sample size is relatively small while the number of features increases greatly, which will increase the probability of making statistical errors [4].

Recently, integrated transcriptomic and quantitative proteomic analyses have been widely used to promote a better understanding of the molecular mechanisms driving biological processes in cells and tissues [5]. Advances in mass-spectrometry (MS) provide an unprecedented opportunity for antibody-independent proteome profiling with approximately 80% of all proteins in major human tissues quantifiable by this technique [6]. By integrating the transcriptomic and proteomic data, the ‘curse of dimensionality’ can be solved through cross-validation in the two levels. Besides, combining datasets from different origins by meta-analysis to extend the sample size and using some machine learning algorithms to select and reduce features could also help solve the ‘curse’ [7].

Due to the difficulty in obtaining atrial tissue from healthy populations, the majority of atrial transcriptomic and proteomic studies of AF used atrial tissue from patients undergoing open-heart surgery with or without AF [8, 9]. By controlling other variables such as the comorbidity, severity of mitral valve disease, age, and sex, analyzing differentially expressed genes (DEGs) or differentially expressed proteins (DEPs) could also help explain the associations between genes expression and this complex disease phenotype. Another commonly applied method is to use samples that are more available in healthy people such as peripheral blood. However, the expression profiles from different cells and tissues could be quite different due to cell/tissue-specific epigenetic regulation mechanism [10]. Hence, we propose to identify feature genes from local atrial tissue as it can directly depict the altered gene expression profiles of atria, and so able to identify the atrial remodeling process of AF.

Here, our objective was to elucidate a more complete understanding of molecular mechanisms underlying AF and to find potential diagnostic and therapeutic targets. The integration of multi-omics data, along with the application of the machine learning approach, vouched for the identification of key pathways and feature genes in AF, which may help to investigate the underlying mechanism of AF and to discover potential diagnostic and therapeutic targets.

## Methods

### Microarray data collection and preprocessing

For the meta-analysis, AF microarray expression data sets were collected from NCBI Gene Expression Omnibus (GEO) database (http://www.ncbi.nlm.nih.gov/geo/). Only microarray data that met the following criteria were included: (1) Data sets were produced by Genome-wide mRNA expression profiling by microarray; (2) The experimental platform was GPL570 (Affymetrix Human Genome U133 Plus 2.0 microarray); (3) Data sets should be gene expression profiles of human atria tissues between AF and sinus rhythm (SR); (4) The minimum number of cases and controls was three. Then, the raw CEL files were downloaded and preprocessed using robust multi array average (RMA) algorithm with ‘affy’ package [11] implemented in R software. The quality of individual samples was assessed using the ‘arrayQualitymetrics’ packages [12]. The outlier samples were excluded if it was detected by array intensity distribution criteria. After that, raw CEL files of the rest samples were preprocessed again using RMA algorithm for background correction, quantile normalization, and summarization.

We then reannotated the probes of GPL570 as it improves accuracy and makes it possible to identify new transcripts. In brief, the probe sequences were downloaded from Affymetrix (affymetrix.com) and were remapped to the human genome (GRCh38 release 99 primary assembly) using the R package ‘Rsubread’ [13]. Then, the chromosomal positions of these probes were matched to the corresponding genome annotation database in Ensembl using the R package ‘GenomicRanges’ [14]. Probe sets that were mapped to > 1 gene were removed to ensure the reliability of the reannotation. The median expression values among all multiple probe IDs were selected to represent the corresponding gene symbol. After that, 19,557 unique genes were retained. The normalized and annotated datasets containing 19,557 rows and 130 columns were used for further meta-analysis.

GSE2240, which contained microarray expression profiles from 10 AF and 20 SR atrial samples, were preprocessed using RMA algorithm and annotated using ‘annotate’ and ‘hgu133a.db’ packages. The median expression values among multiple probe IDs were selected to represent the corresponding gene symbol.

### Microarray meta-analysis using GeneMeta

‘GeneMeta’ Bioconductor package [15] in R was used to perform a microarray meta-analysis of data sets from different ‘origins’. This package is based on the meta-analysis method proposed by Choi et al. [15] using fixed or random effects. In this study, samples regarded as the same ‘origin’ must come from the same tissue (left atria, right atria, etc.) and the same microarray study. The Random effect model (REM) was used [15]. The false discovery rate (FDR) for each gene was obtained with the function “ZscoreFDR” using 1000 permutations. Genes with FDR < 0.05 were considered as DEGs.

### Proteomics study

18 left atrial appendage (LAA) tissue samples were obtained as surgical specimens from patients with mitral stenosis undergoing cardiac surgery at the Second Xiangya Hospital of Central South University, including 9 with chronic AF and 9 with SR. The characteristics of all patients are presented in Table 2. For each clinical group, three samples were mixed into one pooled sample. Qualitative and quantitative proteomic analysis was performed using dimethyl label-coupled high performance liquid chromatography-tandem mass spectrometry (HPLC–MS/MS) and MaxQuant software [16]. Benjamini–Hochberg’s method was used to calculate the FDR. DEPs were identified using a criterion of FDR < 0.1 and fold change > 1.2. The detailed procedure for proteomic study was described in Additional file 1.

### Pathway enrichment analysis

Metascape (https://Metascape.org/) is a web-based portal designed to provide a comprehensive gene list annotation and analysis resource for biologists [17]. It is one of the most effective tools to conducted muti-omics level enrichment analysis. To gain more insights into the biological roles of identified DEGs and DEPs, we conducted pathway enrichment analysis of Gene Ontology biological process (GO BP), Kyoto Encyclopedia of Genes and Genomes (KEGG), Reactome, and Canonical pathway in Metascape tools. By inputting the lists of DEGs and DEPs simultaneously, Metascape can identify commonly-enriched and selectively-enriched pathways from two levels, which enables a comprehensive assessment of the molecular features of the biological process.

### Cross-validation between the transcriptomic and proteomic study

The DEGs and DEPs were further analyzed using VennDiagram to compare and identify the shared genes. To make the selected biomarkers more significant, we only select genes that have consistent expression trends (upregulated or downregulated) between the transcriptomic and proteomic levels for further analysis.

### Feature selection and classification algorithm

The 130 samples involved in the meta-analysis were selected as the training set. The correlation-based feature selection (CFS) method [18] implemented in WEKA solfware [19] was used using the training set to select feature genes. Three popular state-of-the-art supervised classification methods (NB, Naive Bayes; SMO, sequential minimal optimization; and RF, random forest) were used for generating the classification models using WEKA with the default parameter settings [20]. The three algorithms were trained with the training set and their performances were further validated by sixfold cross-validation. The best classifier generated in the training set with the highest accuracy was then validated on the independent test set GSE2240, which contained right atrial appendages samples from 10 AF patients and 20 SR patients undergoing open-heart surgery. The performance of the classifier was evaluated using criteria including precision, recall, F-measure, Matthews correlation coefficient (MCC), AUC (area under receiver operating curve), and auPRC (area under precision-recall curve), true positive rate, false positive rate, and Kappa statistic.

## Results

### Microarray data description and preprocessing

In the transcriptomic meta-analysis study, four microarray data sets were included containing a total of 54 SR and 79 AF paired atrial samples (Table 1) from patients with valvular heart disease. The included raw CEL files were pre-processed and quality control analysis of the data sets (after normalization) led to the removal of 3 samples including GSM1005420, GSM3182694, and GSM3182707. After removing the outliers and reprocessing, the normalized data sets consisting of 130 samples were taken for further meta-analysis approach.

**Table 1:** Characteristics of publically available GEO data sets used in the microarray meta-analysis

Accession number	Organism	Platform	Number of samples (SR/AF)	Origin
GSE41177	Homo sapiens	Affymetrix Human Genome U133 Plus 2.0	Left atrial appendage: 3/16	1
			Left atrial junction: 3/16	2
GSE79768	Homo sapiens	Affymetrix Human Genome U133 Plus 2.0	Left atrial specimen: 6/7	3
			Right atrial specimen:6/7	4
GSE115574	Homo sapiens	Affymetrix Human Genome U133 Plus 2.0	Left atrial tissue: 15/14	5
			Right atrial tissue: 16/14	6
GSE14975	Homo sapiens	Affymetrix Human Genome U133 Plus 2.0	Left atrial appendage: 5/5	7

### Identification of DEGs

As shown in Table 1, we only considered samples from the same study and the same tissue as the same ‘origin’, which led to a total of 7 different origins. We then performed a meta-analysis by using the R package ‘GeneMeta’ and DEGs were detected by comparing the differential expression levels between the AF and SR group. The results identified 863 genes as DEGs (FDR < 0.05; 485 up-regulated: z-score > 0; 378 down-regulated: z-score < 0) (Additional file 2).

### Results of proteomic study

**Fig. 1:**
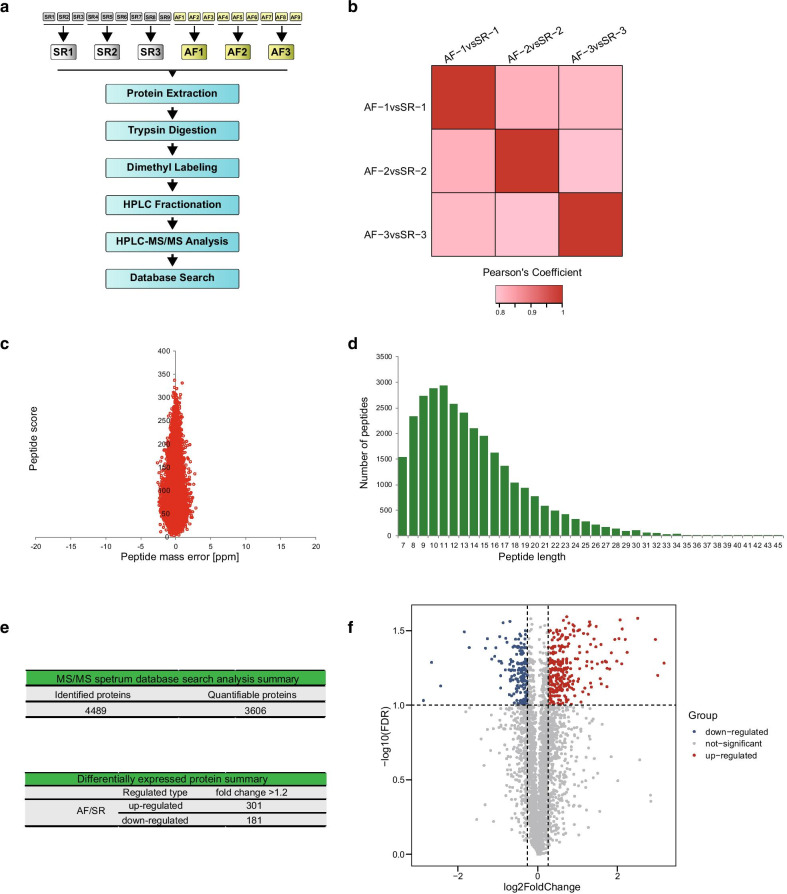
Quantitative proteomic analysis of AF and SR tissue samples. **a** Experimental process; **b** reproducibility of the quantitative proteomic analysis; **c** QC validation of MS data. Mass error indicates the distribution of all identified peptides. **d** Peptide length distribution identified by quantitative proteomic analysis; **e** identified and quantified proteins; **f** Volcano plot of differentiated expressed proteins

The characteristics of the patients included in the proteomic study were balanced between the two groups, except for the left atrial (LA) size (Table 2). Figure 1a shows the procedure of the proteomic study. Pearson’s correlation analysis indicated good repeatability between the samples (Fig. 1b). The mass accuracy of the MS data met the requirement (Fig. 1c) and the distribution of peptides’ length agreed with the properties of tryptic peptides (Fig. 1d). In total, we identified 4489 proteins including 3606 quantifiable proteins (Fig. 1e). Proteins with FDR < 0.1 and fold change > 1.2 were considered significant, which led to the identification of 482 DEPs (301 upregulated and 181 downregulated) (Fig. 1e, f) (Additional file 3).

**Table 2:** Characteristics of the patients with mitral stenosis involved in the proteomic study

	SR (n = 9)	AF (n = 9)	*p*
Male (n, %)	5 (55.6%)	4 (44.4%)	1
Age (year)	50.5 ± 6.5	55.5 ± 9.0	0.195
BMI (kg/m^2^)	22.2 ± 2.0	22.7 ± 1.8	0.489
Hypertension (n, %)	4 (44.4%)	6 (66.7%)	0.637
Hemoglobin (g/L)	135.7 ± 16.8	128.1 ± 22.4	0.546
WBC (109/L)	6.2 ± 2.1	6.7 ± 1.6	0.546
Platelet (109/L)	225.8 ± 86.4	205.2 ± 44.0	0.931
ALT (u/L)	18.7 ± 11.4	19.6 ± 7.7	0.666
AST (u/L)	20.2 ± 4.9	24.8 ± 12.4	0.605
ALB (g/L)	37.4 ± 1.9	39.1 ± 4.2	0.489
Serum creatinine (umol/L)	64.5 ± 21.0	69.1 ± 15.6	0.222
NT-proBNP (pg/mL)	161.4 ± 77.7	201.8 ± 138.7	0.546
Fasting blood glucose (mmol/L)	5.0 ± 0.4	5.2 ± 0.4	0.489
Total cholesterol (mmol/L)	4.6 ± 0.5	4.3 ± 0.5	0.269
RA size (mm)	33.0 ± 4.3	33.0 ± 4.3	0.796
LA size (mm)*	37.9 ± 3.1	49.4 ± 8.0	0.001
RV size (mm)	30.1 ± 4.9	33.8 ± 8.1	0.489
LV size (mm)	46.9 ± 10.6	54.1 ± 10.8	0.161
EF (%)	62.9 ± 8.6	61.3 ± 8.8	0.711
Mitral valve area (cm^2^)	1.8 ± 0.3	1.9 ± 0.3	0.746
NYHA class (I/II)	9/0	6/3	

*p < 0.05

### Pathway enrichment analysis and visualization

**Fig. 2:**
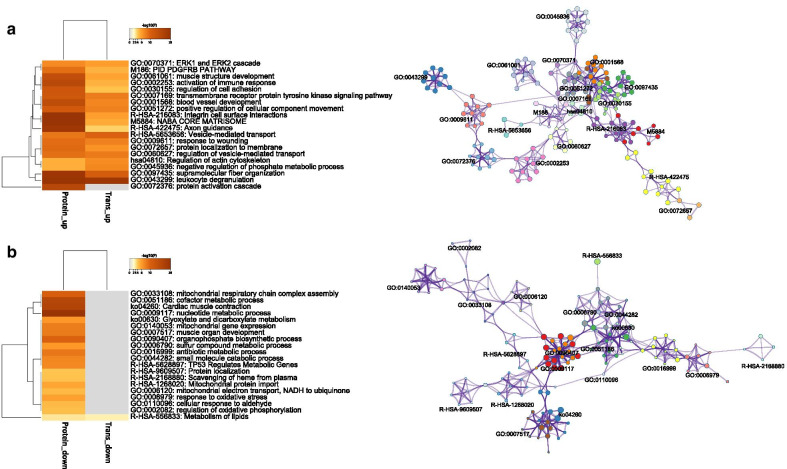
Pathway enrichment analysis. **a** Top 20 clusters with the smallest *p* value of upregulated mRNAs/proteins; **b** Top 20 clusters with the smallest *p* value of downregulated mRNAs/proteins right. The right part displays the network of selected enriched terms. Each term is represented by a circle node, where its size is proportional to the number of input genes that fall into that term, and its color represents its cluster identity (i.e., nodes of the same color belong to the same cluster). Terms with a similarity score > 0.3 are linked by an edge (the thickness of the edge represents the similarity score)

Pathway enrichment analysis helps researchers gain mechanistic insight into gene lists generated from genome-scale (omics) experiments. This method identifies biological pathways that are enriched in a gene list more than would be expected by chance. Metascape helps to integrate different omics data such as genomics, transcriptomics, and proteomics, which enables a comprehensive understanding of a biological process. Unlike other methods, Metascape clusters enriched terms into non-redundant groups that will be critical for informing future studies. We visualized the top 20 clusters and chose the most significant (lowest *p* value) term within each of the 20 clusters to represent the cluster. For the upregulated proteins and mRNAs, most of the top 20 clusters (19) were enriched in both protein and mRNA levels, which highly suggested the importance of these pathways in AF pathogenesis (Fig. 2a). While for the down-regulated ones, the top 20 clusters were mainly involved in energy metabolism-related pathways, and these pathways were only enriched in the protein level (Fig. 2b). To further capture the relationships between the terms, we selected a subset of representative terms from each of the 20 clusters (up to the 10 best scoring terms) and convert them into a network layout which was visualized within Cytospace (Fig. 2, right part).

### Cross-validation

**Fig. 3:**
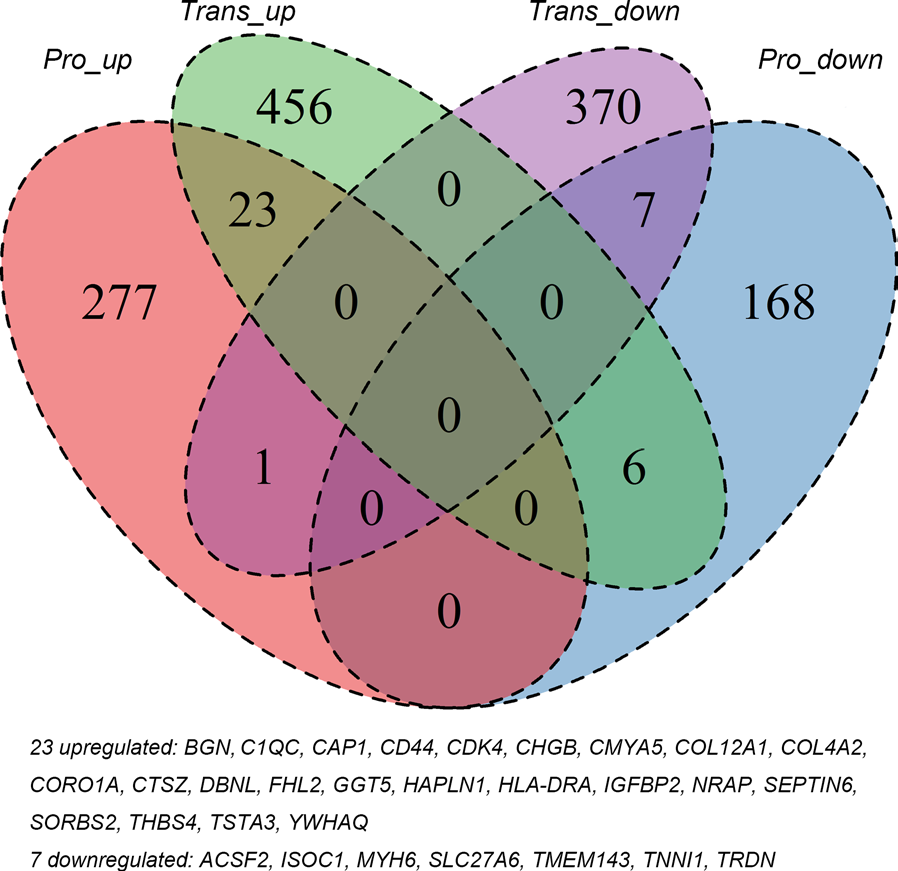
Venn diagram of DEGs and DEPs

To make the selected biomarkers more significant, we only select genes that have consistent expression trends (upregulated or downregulated) between the transcriptomic and proteomic levels for further analysis. As VennDiagram showed (Fig. 3), 23 up-regulated genes/proteins, and 7 down-regulated genes/proteins were identified to have consistent trends from two-level. These 30 genes/proteins were considered important biomarkers for AF.

### Performance evaluation of AF classifier

After feature selection using training set, the number of features reduced from 30 to 10 including CD44, CHGB, FHL2, GGT5, IGFBP2, NRAP, SEPTIN6, YWHAQ, TNNI1, and TRDN. After removing the bath effect using ‘sva’ packages in the R solfware, the expression values of these 10 features were used to generate classifiers with three supervised machine learning algorithms—NB, SMO, and RF, based on the training set. We first conducted sixfold cross-validation to classify AF and SR samples. All classifiers performed well with a precision of 86.9% for NB, 86.3% for SMO, and 76.8% for RF (Table 3). Base on a comprehensive evaluation of precision and other measures, the NB classifier performed best and the constructed NB classifier using the whole training set was further evaluated in the independent test set. Among the 30 atrial samples, 24 of them (80%) were correctly classified. The performance criteria including precision, recall, F-measure, MCC, AUC, auPRC, true positive rate, false positive rate, and Kappa statistic were 87.5%, 0.8, 0.805, 0.661, 0.995, 0.995, 0.8, 0.1, and 0.609, respectively. Therefore, the overall measures of high accuracy confirmed the efficacy of the classifier to distinguish AF from SR samples, which further proved that the 10 gene feature are important biomarkers for AF.

**Table 3:** Performance of different prediction models generated by sixfold cross-validation on the training data set

Classifier	Precision	Recall	F-Measure	MCC	AUC	auPRC	TP rate	FP rate	Kappa statistic
NB	0.869	0.869	0.869	0.729	0.925	0.920	0.869	0.143	0.728
SMO	0.863	0.862	0.862	0.715	0.860	0.814	0.862	0.142	0.715
RF	0.768	0.769	0.768	0.518	0.887	0.881	0.769	0.259	0.516

*NB* Naive Bayes, *SMO* sequential minimal optimization, *RF* random forest, *MCC* Matthews correlation coefficient, *AUC* area under receiver operating curve, *auPRC* area under precision recall curve, *TP* true positive, *FP* false positive

## Discussion

To our knowledge, this is the first integrated transcriptomic and proteomic analysis of human AF atrial tissue, and the first to identify feature genes of AF using machine learning approach. Previous transcriptomic studies have provided insights into the pathogenesis of AF [21, 22]. However, these experiments are generally analyzed through a single data source or restricted to a fewer sample which can lead to biological and technical biases. Thus, the microarray meta-analysis was used in this study to integrate four microarray data sets of AF from GEO which led to the identification of 863 DEGs. To elucidate a more complete understanding of AF pathogenesis, we also conducted a proteomic study of local atrial tissue which identified 482 DEPs.

Pathway enrichment analysis can help to characterize physiological and functional changes associated with the changes in mRNA and protein expression in AF atrial tissues. For the upregulated mRNAs or proteins, the top 19 scoring items were enriched in both transcriptomic and proteomic levels, which vouched for the importance and significance of these pathways. Some of the items, such as ‘PDGFRB PATHWAY’, ‘activation of immune response’, ‘muscle structure development’, ‘regulation of actin cytoskeleton’, and ‘leukocyte degranulation’, have been proved to play key roles in AF progression [3, 23]. For the downregulated mRNAs or proteins, the top 19 scoring items were only enriched in the proteomic level, and these pathways were mainly involved in metabolism regulation, such as ‘mitochondrial respiratory chain complex assembly’, ‘TP53 regulates metabolic genes’, and ‘response to oxidative stress’. Besides, the ‘Metabolism of lipids’ pathway was enriched in two levels. These are in accord with the recent studies which highlighted the role of metabolic remodeling in AF [24–26]. The reason why these pathways are only identified in the protein level may be caused by some post-transcriptional and translational regulations.

After cross-validation between the two omics data. We identified 30 genes or proteins with the same trends between two levels. To make the selected features more significant and informative, the machine learning CFS feature selection method was adopted in the training set which led to the final 10 features, wherein 8 are upregulated (CD44, CHGB, FHL2, GGT5, IGFBP2, NRAP, SEPTIN6, YWHAQ) and 2 are downregulated (TNNI1, TRDN). The NB classifier base on the expression values of these features in the training set can classify AF and SR samples with a precision of 87.5% and AUC of 0.995 in the independent test set.

Some of these feature genes have been reported to be associated with AF or its related pathogenesis. The CD44 related pathways including CD44/STAT3 and CD44/NOX4 signaling pathways can lead to atrial fibrosis [27] and Ca^2+^-handling abnormalities [28] during AF. Secretogranin-1 (CHGB) presents in the secretory granules in atrial myoendocrine cells and is co-localized with atrial natriuretic peptide (ANP) while CHGB genetic variation results in oxidative stress [29] and hypertension [30]. The four and a half LIM domains protein 2 (FHL2) is a component of the hypertrophic response and is found to be protective in cardiac hypertrophic through inhibiting MAPK/ERK signaling [31]. MAPK has been proved to function in AF context by mediating oxidative stress [32, 33], epicardial adipose tissue remodeling [34], atrial fibrosis [35], load-induced hypertrophic response [36], and ionic channel remodeling [37]. Gamma-glutamyltransferase-5 (GGT5) is confirmed to be closely associated with immune cell activation [38] and oxidative stress [39, 40] and can be a potential biomarker of myocardial infarction [41]. Insulin-like growth factor-binding protein 2 (IGFBP2) belongs to the insulin-like growth factor-binding protein (IGFBP) family. Two recent studies observed a higher hazard of incident AF associated with higher mean levels of plasma IGFBP1 protein [42] and IGFBP3 protein [43]. Nebulin related anchoring protein (NRAP) is present in myofibril precursors during myofibrillogenesis and thought to be involved in myofibril assembly [44], and its genetic variance is associated with cariomyopathy [45]. Septin-6 (SEPTIN6) is invovled in extracellular matrix remodeling [46]. 14–3-3 protein theta (YWHAQ) is a gene in the P53 network and has been shown to promote apoptosis directly upon genotoxic stress [47]. Another proteomic also identified YWHAQ as an important biomarker in AF [47]. TNNI1 encodes a troponin-I protein that is the dominant form of troponin-I expressed in the fetal/neonatal/infant heart, and its participants in AF remains unknown. Triadin (TRDN) is a stable subunit of the ryanodine receptor 2 (RyR2) and is involved in the regulation of Ca^2+^ release [48]. The loss or dysfunction of RyR2 stable subunits was demonstrated to cause the occurrence of spontaneous calcium elevation in AF atrial cells [49]. Our present study further proved and emphasized the importance of these markers.

There are some limitations to the current study. Firstly, the number of samples included in the microarray meta-analysis remains relatively small (n = 130), which is caused by the limited number of available studies in the GEO database. Secondly, there is no corresponding clinical information of the samples, we were not able to make a prognostic analysis of these biomarkers. Third, the samples used in the transcriptomic and proteomic studies came from patients with valvular heart disease. This is due to the difficulty in acquiring atrial samples from healthy cohorts. The psychophysiology of AF in patients with valvular heart disease may have some differences from those with non-valvular AF. We recommend further study to identify gene expression profiles using atrial samples from non-valvular AF patients and healthy donors. Finally, the transcriptomic and proteomic can only indicate the potential causes for a phenotypic response, but they cannot predict what will happen at the next level. Thus, one should consider the metabolomic that provides a functional view of an organism as determined by the sum of its genes, RNA, proteins, and environmental factors [50]. Nonetheless, the integrated analysis of multi-omics data along with the machine learning method makes sure the selected genes as important features for AF. Further studies are needed to clarify their functions in AF pathogenesis.

## Conclusions

In conclusion, the current study identified a list of significantly dysregulated feature genes associated with AF using a multi-omics analysis. The machine learning feature selection identified 10 feature genes. Naive Bayes prediction model built in the training set using the expression profiles of 10 features performed accurately and reliably classified AF from SR samples in the independent test set. These findings could provide novel insight into the pathogenesis of AF and suggested that the feature genes might be diagnostic and therapeutic targets for AF.

## Supplementary Information

**Additional file 1.** Detailed procedure of the proteomic study.

**Additional file 2.** Results of the microarray meta-analysis.

**Additional file 3.** Results of the proteomic study.

## Data Availability

The microarray datasets analyzed during the present study are available from the Gene Expression Omnibus repository (https://www.ncbi.nlm.nih.gov/geo). The accession numbers were GSE41177, GSE79768, GSE115574, GSE14975, and GSE2240. Results of the proteomic study were submitted as supplementary material.

## Abbreviations

AF: Atrial fibrillation
GEO: Gene Expression Omnibus
MS: Mass spectrometry
DEPs: Differentially expressed proteins
SR: Sinus rhythm
RMA: Robust multiarray average
REM: Random effect model
FDR: False discovery rate
DEGs: Differentially expressed genes
LAA: Left atrial appendage
HPLC–MS/MS: High-performance liquid chromatography-tandem mass spectrometry
GO BP: Gene Ontology biological process
KEGG: Kyoto Encyclopedia of Genes and Genomes
CFS: Correlation-based feature selection
NB: Naive Bayes
SMO: Sequential minimal optimization
RF: Random forest
AUC: Area under receiver operating curve
MCC: Matthews correlation coefficient
auPRC: Area under precision-recall curve

## References

[1] Kirchhoff P, Benussi S, Kotecha D (2016). 2016 ESC Guidelines for the management of atrial fibrillation developed in collaboration with EACTS. Eur Heart J.

[2] Chugh SS, Havmoeller R, Narayanan K, Singh D, Rienstra M, Benjamin EJ, Gillum RF, Kim YH, McAnulty JH, Zheng ZJ, Forouzanfar MH, Naghavi M, Mensah GA, Ezzati M, Murray CJ (2014). Worldwide epidemiology of atrial fibrillation: a Global Burden of Disease 2010 Study. Circulation.

[3] Schotten U, Verheule S, Kirchhof P, Goette A (2011). Pathophysiological mechanisms of atrial fibrillation: a translational appraisal. Physiol Rev.

[4] Loris N, Sheryl B, Alessandra L (2012). Combining multiple approaches for gene microarray classification. Bioinformatics.

[5] Ghazalpour A, Bennett B, Petyuk VA, Orozco L, Hagopian R, Mungrue IN, Farber CR, Sinsheimer J, Kang HM, Furlotte N, Park CC, Wen PZ, Brewer H, Weitz K, Camp DG, Pan C, Yordanova R, Neuhaus I, Tilford C, Siemers N, Gargalovic P, Eskin E, Kirchgessner T, Smith DJ, Smith RD, Lusis AJ (2011). Comparative analysis of proteome and transcriptome variation in mouse. PLoS Genet.

[6] Kim M-S, Pinto SM, Getnet D, Nirujogi RS, Manda SS, Chaerkady R, Madugundu AK, Kelkar DS, Isserlin R, Jain S, Thomas JK, Muthusamy B, Leal-Rojas P, Kumar P, Sahasrabuddhe NA, Balakrishnan L, Advani J, George B, Renuse S, Selvan LDN, Patil AH, Nanjappa V, Radhakrishnan A, Prasad S, Subbannayya T, Raju R, Kumar M, Sreenivasamurthy SK, Marimuthu A, Sathe GJ, Chavan S, Datta KK, Subbannayya Y, Sahu A, Yelamanchi SD, Jayaram S, Rajagopalan P, Sharma J, Murthy KR, Syed N, Goel R, Khan AA, Ahmad S, Dey G, Mudgal K, Chatterjee A, Huang T-C, Zhong J, Wu X, Shaw PG, Freed D, Zahari MS, Mukherjee KK, Shankar S, Mahadevan A, Lam H, Mitchell CJ, Shankar SK, Satishchandra P, Schroeder JT, Sirdeshmukh R, Maitra A, Leach SD, Drake CG, Halushka MK, Prasad TSK, Hruban RH, Kerr CL, Bader GD, Iacobuzio-Donahue CA, Gowda H, Pandey A (2014). A draft map of the human proteome. Nature.

[7] Ramasamy A, Mondry A, Holmes CC, Altman DG (2008). Key issues in conducting a meta-analysis of gene expression microarray datasets. PLoS Med.

[8] Steenman M (2020). Insight into atrial fibrillation through analysis of the coding transcriptome in humans. Biophys Rev.

[9] Sühling M, Wolke C, Scharf C, Lendeckel U (2018). Proteomics and transcriptomics in atrial fibrillation. Herzschrittmachertherapie Elektrophysiologie.

[10] Roselli C, Rienstra M, Ellinor PT (2020). Genetics of atrial fibrillation in 2020: GWAS, genome sequencing, polygenic risk, and beyond. Circ Res.

[11] Gautier L, Cope L, Bolstad BM, Irizarry RA (2004). affy-analysis of Affymetrix GeneChip data at the probe level. Bioinformatics.

[12] Audrey K, Robert G, Wolfgang H (2008). arrayQualityMetrics—a bioconductor package for quality assessment of microarray data. Bioinformatics.

[13] Liao Y, Smyth G, Shi W (2019). The R package Rsubread is easier, faster, cheaper and better for alignment and quantification of RNA sequencing reads. Nucleic Acids Res.

[14] Lawrence M, Huber W, Pagès H, Aboyoun P, Carlson M, Gentleman R, Morgan M, Carey V (2013). Software for computing and annotating genomic ranges. PLoS Comput Biol.

[15] Choi JK, Yu U, Kim S, Yoo OJ (2003). Combining multiple microarray studies and modeling interstudy variation. Bioinformatics (Oxford, England).

[16] Cox J, Mann M (2008). MaxQuant enables high peptide identification rates, individualized p.p.b.-range mass accuracies and proteome-wide protein quantification. Nat Biotechnol.

[17] Zhou Y, Zhou B, Pache L, Chang M, Khodabakhshi AH, Tanaseichuk O, Benner C, Chanda SK (2019). Metascape provides a biologist-oriented resource for the analysis of systems-level datasets. Nat Commun.

[18] Lei Y, Liu H. Feature selection for high-dimensional data: a fast correlation-based filter solution, machine learning. In: Proceedings of the twentieth international conference (ICML 2003), August 21–24, 2003, Washington, DC, USA, 2003.

[19] Hall M, Frank E, Holmes G, Pfahringer B, Reutemann P, Witten IH (2009). The WEKA data mining software: an update. SIGKDD Explor Newsl.

[20] Naorem LD, Muthaiyan M, Venkatesan A (2019). Integrated network analysis and machine learning approach for the identification of key genes of triple-negative breast cancer. J Cell Biochem.

[21] Barth AS, Merk S, Arnoldi E, Zwermann L, Kloos P, Gebauer M, Steinmeyer K, Bleich M, Kääb S, Hinterseer M (2005). Reprogramming of the human atrial transcriptome in permanent atrial fibrillation: expression of a ventricular-like genomic signature. Circ Res.

[22] Deshmukh A, Barnard J, Sun H, Newton D, Castel L, Pettersson G, Johnston D, Roselli E, Gillinov AM, McCurry K, Moravec C (2015). Left atrial transcriptional changes associated with atrial fibrillation susceptibility and persistence. Circ Arrhythm Electrophysiol.

[23] Liu Y, Shi Q, Ma Y, Liu Q (2018). The role of immune cells in atrial fibrillation. J Mol Cell Cardiol.

[24] Opacic D, van Bragt KA, Nasrallah HM, Schotten U, Verheule S (2016). Atrial metabolism and tissue perfusion as determinants of electrical and structural remodelling in atrial fibrillation. Cardiovasc Res.

[25] Liu Y, Bai F, Liu N, Ouyang F, Liu Q (2019). The Warburg effect: a new insight into atrial fibrillation. Clin Chim Acta Int J Clin Chem.

[26] Bai F, Tu T, Qin F, Ma Y, Liu N, Liu Y, Liao X, Zhou S, Liu Q (2019). Quantitative proteomics of changes in succinylated proteins expression profiling in left appendages tissue from valvular heart disease patients with atrial fibrillation. Clin Chim Acta.

[27] Chang SH, Yeh YH, Lee JL, Hsu YJ, Kuo CT, Chen WJ (2017). Transforming growth factor-beta-mediated CD44/STAT3 signaling contributes to the development of atrial fibrosis and fibrillation. Basic Res Cardiol.

[28] Chen WJ, Chang SH, Chan YH, Lee JL, Lai YJ, Chang GJ, Tsai FC, Yeh YH (2019). Tachycardia-induced CD44/NOX4 signaling is involved in the development of atrial remodeling. J Mol Cell Cardiol.

[29] Rao F, Zhang K, Khandrika S, Mahata M, Fung MM, Ziegler MG, Rana BK, O'Connor DT (2010). Isoprostane, an “intermediate phenotype” for oxidative stress heritability, risk trait associations, and the influence of chromogranin B polymorphism. J Am Coll Cardiol.

[30] Zhang K, Rao F, Wang L, Rana BK, Ghosh S, Mahata M, Salem RM, Rodriguez-Flores JL, Fung MM, Waalen J, Tayo B, Taupenot L, Mahata SK, O'Connor DT (2010). Common functional genetic variants in catecholamine storage vesicle protein promoter motifs interact to trigger systemic hypertension. J Am Coll Cardiol.

[31] Liang Y, Bradford WH, Zhang J, Sheikh F (2018). Four and a half LIM domain protein signaling and cardiomyopathy. Biophys Rev.

[32] Rochette L, Lorin J, Zeller M, Guilland JC, Lorgis L, Cottin Y, Vergely C (2013). Nitric oxide synthase inhibition and oxidative stress in cardiovascular diseases: possible therapeutic targets?. Pharmacol Ther.

[33] Liang X, Zhang Q, Wang X, Yuan M, Zhang Y, Xu Z, Li G, Liu T (2018). Reactive oxygen species mediated oxidative stress links diabetes and atrial fibrillation. Mol Med Rep.

[34] Suffee N, Moore-Morris T, Farahmand P, Rucker-Martin C, Dilanian G, Fradet M, Sawaki D, Derumeaux G, LePrince P, Clement K, Dugail I, Puceat M, Hatem SN (2017). Atrial natriuretic peptide regulates adipose tissue accumulation in adult atria. Proc Natl Acad Sci USA.

[35] Fan J, Zou L, Cui K, Woo K, Du H, Chen S, Ling Z, Zhang Q, Zhang B, Lan X, Su L, Zrenner B, Yin Y (2015). Atrial overexpression of angiotensin-converting enzyme 2 improves the canine rapid atrial pacing-induced structural and electrical remodeling. Basic Res Cardiol.

[36] Kerkela R, Ilves M, Pikkarainen S, Tokola H, Ronkainen VP, Majalahti T, Leppaluoto J, Vuolteenaho O, Ruskoaho H (2011). Key roles of endothelin-1 and p38 MAPK in the regulation of atrial stretch response, American journal of physiology. Regul Integr Comparat Physiol.

[37] Cheng W, Zhu Y, Wang H (2016). The MAPK pathway is involved in the regulation of rapid pacing-induced ionic channel remodeling in rat atrial myocytes. Mol Med Rep.

[38] Lu E, Wolfreys FD, Muppidi JR, Xu Y, Cyster JG (2019). S-Geranylgeranyl-L-glutathione is a ligand for human B cell-confinement receptor P2RY8. Nature.

[39] Li W, Wu ZQ, Zhang S, Cao R, Zhao J, Sun ZJ, Zou W (2016). Augmented expression of gamma-glutamyl transferase 5 (GGT5) impairs testicular steroidogenesis by deregulating local oxidative stress. Cell Tissue Res.

[40] Dhingra R, Gona P, Wang TJ, Fox CS, D'Agostino RB, Vasan RS (2010). Serum gamma-glutamyl transferase and risk of heart failure in the community. Arterioscler Thromb Vasc Biol.

[41] Sharma A, Ghatge M, Mundkur L, Vangala R (2016). Translational informatics approach for identifying the functional molecular communicators linking coronary artery disease, infection and inflammation. Mol Med Rep.

[42] Staerk L, Preis SR, Lin H, Lubitz SA, Ellinor PT, Levy D, Benjamin EJ, Trinquart L (2020). Protein biomarkers and risk of atrial fibrillation: the FHS. Circ Arrhythm Electrophysiol.

[43] Busch M, Kruger A, Gross S, Ittermann T, Friedrich N, Nauck M, Dorr M, Felix SB (2019). Relation of IGF-1 and IGFBP-3 with prevalent and incident atrial fibrillation in a population-based study. Heart Rhythm.

[44] Bang ML, Chen J (2015). Roles of nebulin family members in the heart. Circ J Off J Jpn Circ Soc.

[45] Vasilescu C, Ojala TH, Brilhante V, Ojanen S, Hinterding HM, Palin E, Alastalo TP, Koskenvuo J, Hiippala A, Jokinen E, Jahnukainen T, Lohi J, Pihkala J, Tyni TA, Carroll CJ, Suomalainen A (2018). Genetic basis of severe childhood-onset cardiomyopathies. J Am Coll Cardiol.

[46] Collins KB, Kang H, Matsche J, Klomp JE, Rehman J, Malik AB, Karginov AV (2020). Septin2 mediates podosome maturation and endothelial cell invasion associated with angiogenesis. J Cell Biol..

[47] Vazquez A, Grochola LF, Bond EE, Levine AJ, Taubert H, Müller TH, Würl P, Bond GL (2010). Chemosensitivity profiles identify polymorphisms in the p53 network genes 14-3-3tau and CD44 that affect sarcoma incidence and survival. Can Res.

[48] Franzini-Armstrong C, Protasi F, Tijskens P (2005). The assembly of calcium release units in cardiac muscle. Ann N Y Acad Sci.

[49] Zhang JC, Wu HL, Chen Q, Xie XT, Zou T, Zhu C, Dong Y, Xiang GJ, Ye L, Li Y, Zhu PL (2018). Calcium-mediated oscillation in membrane potentials and atrial-triggered activity in atrial cells of Casq2(R33Q/R33Q) mutation mice. Front Physiol.

[50] Mercuro G, Bassareo P, Deidda M, Cadeddu C, Barberini L, Atzori L (2011). Metabolomics: a new era in cardiology?. J Cardiovasc Med (Hagerstown, Md.).

