# Supplementary material for: Integrative transcriptomic, proteomic, and machine learning approach to identifying feature genes of atrial fibrillation using atrial samples from patients with valvular heart disease
Source: BMC Cardiovasc Disord. 2021 Jan 28;21:52. doi: 10.1186/s12872-020-01819-0 (PMC7842070; doi:10.1186/s12872-020-01819-0)
Supplement: Supplementary file 1 — Additional file 1. Detailed procedure of the proteomic study. [file 12872_2020_1819_MOESM1_ESM.docx]

The detailed procedure of the proteomic study

1. **Ethical approval**

This study was approved by the Ethics Committee of the Second Xiangya Hospital of Central South University. The research was carried out following the World Medical Association Declaration of Helsinki. Informed written consent was obtained from all patients.

1. **Patients and samples**

18 left atrial appendage (LAA) tissue samples were obtained as surgical specimens from patients with mitral stenosis undergoing cardiac surgery at the Second Xiangya Hospital of Central South University, including 9 with chronic AF and 9 with sinus rhythm (SR)(without a history of AF). Tissue samples were transferred to liquid nitrogen immediately and stored at −80 °C. All patients were divided into the SR group and chronic AF group according to clinical symptoms and electrocardiogram/dynamic electrocardiogram. Chronic AF was defined as long-standing persistent AF (lasts more than 12 months) and permanent AF. All patients received routine transthoracic ultrasonography and plasma glucose/lipid analysis before surgery, and their body mass index (BMI) was calculated. Patients > 50 years old were routinely excluded from coronary artery disease.

1. **Protein extraction**

When analyzing the proteomic profile, we mixed three tissues as one pooled sample. Then we have three repeating mixed samples in each clinical group. The frozen LAA samples were grinded by liquid nitrogen into cell powder and then lysed using lysis buffer (8 M urea, 1% protease inhibitor Cocktail, and 2 mM ethylene diamine tetraacetic acid). The remaining debris was removed by centrifugation at 12,000 g at 4 °C for 10 min. The protein concentration was determined with BCA kit.

1. **Trypsin digestion**

The protein solution was reduced with 5 mM dithiothreitol for 30 min at 56 °C and alkylated with 11 mM iodoacetamide for 15 min at room temperature in darkness. The protein sample was then diluted by adding 100 mM TEAB to urea concentration less than 2M. Finally, trypsin was added at 1:50 trypsin-to-protein mass ratio for the first digestion overnight and 1:100 trypsin-to-protein mass ratio for a second 4 h-digestion.

1. **Dimethyl labeling**

Following trypsin digestion, The trypsinized peptides were desalted with Strata X C18 (Phenomenex) and then dried using the vacuum freezing method. Then we dissolve the peptide using TEAB with a concentration of 0.5M and label the peptide according to the instructions of the dimethyl kit.

1. **HPLC fractionation**

The tryptic peptides were fractionated into fractions by high pH reverse-phase HPLC using Agilent 300Extend C18 column (5 μm particles, 4.6 mm ID, 250 mm length). Briefly, peptides were first separated with a gradient of 8% to 32% acetonitrile (pH 9.0) over 60 min into 60 fractions. Then, the peptides were combined into 18 fractions and dried by vacuum centrifuging.

1. **HPLC-MS/MS analysis**

The tryptic peptides were dissolved in 0.1% formic acid (solvent A), directly loaded onto a home-made reversed-phase analytical column (15-cm length, 75 μm i.d.). The gradient was comprised of an increase from 6% to 23% solvent B (0.1% formic acid in 98% acetonitrile) over 26 min, 23% to 35% in 8 min and climbing to 80% in 3 min then holding at 80% for the last 3 min, all at a constant flow rate of 400 nL/min on an EASY-nLC 1000 UPLC system. The peptides were subjected to NSI source followed by tandem mass spectrometry (MS/MS) in Q ExactiveTM Plus (Thermo) coupled online to the UPLC. The electrospray voltage applied was 2.0 kV. The m/z scan range was 350 to 1800 for full scan, and intact peptides were detected in the Orbitrap at a resolution of 70,000. Peptides were then selected for MS/MS using NCE setting as 28 and the fragments were detected in the Orbitrap at a resolution of 17,500. A data-dependent procedure that alternated between one MS scan followed by 20 MS/MS scans with 15.0s dynamic exclusion. Automatic gain control (AGC) was set at 5E4. The fixed first mass was set as 100 m/z.

1. **Database search**

The resulting MS/MS data were processed using Maxquant search engine (v.1.5.2.8). Tandem mass spectra were searched against concatenated with reverse decoy database. Trypsin/P was specified as cleavage enzyme allowing up to 2 missing cleavages. The mass tolerance for precursor ions was set as 20 ppm in First search and 5 ppm in Main search, and the mass tolerance for fragment ions was set as 0.02 Da. Carbamidomethyl on Cys was specified as fixed modification and oxidation on Met was specified as variable modifications. FDR was adjusted to < 1% and the minimum score for peptides was set > 40.
